# PyPore3D: An Open Source Software Tool for Imaging Data Processing and Analysis of Porous and Multiphase Media

**DOI:** 10.3390/jimaging8070187

**Published:** 2022-07-07

**Authors:** Amal Aboulhassan, Francesco Brun, George Kourousias, Gabriele Lanzafame, Marco Voltolini, Adriano Contillo, Lucia Mancini

**Affiliations:** 1 Elettra-Sincrotrone Trieste S.C.p.A., 34149 Trieste, Italy; marco.voltolini@unimi.it (M.V.); adriano.contillo@elettra.eu (A.C.); lucia.mancini@zag.si (L.M.); 2 The Abdus Salam International Centre for Theoretical Physics ICTP, I-34151 Trieste, Italy; 3 Department of Engineering and Architecture, University of Trieste, 34127 Trieste, Italy; fbrun@units.it; 4 Department of Biological, Geological and Environmental Sciences, University of Catania, 95131 Catania, Italy; gabriele.lanzafame@unict.it; 5 Department of Materials, Slovenian National Building and Civil Engineering Institute, 1000 Ljubljana, Slovenia

**Keywords:** tomographic 3D/4D imaging data, image processing and analysis, open source software, Python

## Abstract

In this work, we propose the software library PyPore3D, an open source solution for data processing of large 3D/4D tomographic data sets. PyPore3D is based on the Pore3D core library, developed thanks to the collaboration between Elettra Sincrotrone (Trieste) and the University of Trieste (Italy). The Pore3D core library is built with a distinction between the User Interface and the backend filtering, segmentation, morphological processing, skeletonisation and analysis functions. The current Pore3D version relies on the closed source IDL framework to call the backend functions and enables simple scripting procedures for streamlined data processing. PyPore3D addresses this limitation by proposing a full open source solution which provides Python wrappers to the the Pore3D C library functions. The PyPore3D library allows the users to fully use the Pore3D Core Library as an open source solution under Python and Jupyter Notebooks PyPore3D is both getting rid of all the intrinsic limitations of licensed platforms (e.g., closed source and export restrictions) and adding, when needed, the flexibility of being able to integrate scientific libraries available for Python (SciPy, TensorFlow, etc.).

## 1. Introduction

Recently, the production of three dimensional (3D) images obtained by X-ray computed tomography (CT) experiments is gaining increasing interest by the scientific community. This is due to the big potential of this technique for the nondestructive characterisation of the internal microstructure of opaque objects.

Over the last decade, the advances in both synchrotron light-based sources, as well as desktop CT imaging equipment, have lead to producing large 3D data sets while the acquisition time of synchrotron-based CT scans has significantly been reduced [1]. This is leading to producing high-resolution 3D images at the micron and sub-micron scale, which provide large potential for understanding the internal structure of microstructures. However, since the reconstruction and subsequent data processing steps are still relatively more time-consuming, this provides a challenging bottleneck in the relevant data reduction that motivated the need for developing efficient techniques for data processing and analysis.

As far as data analysis software is concerned, performance optimisation requires working on three aspects: usability, efficiency and availability. From a software development perspective, till now, low-level languages, such as C, are the most favoured when it comes to performance. Furthermore, GPU computing (e.g., the CUDA-based platform) in recent years has enabled faster processing. However, being based on low-level programming languages, it is still challenging to program. Both approaches need sophisticated programming skills, which are obviously not suitable for the scientific community who prefer to rather focus on fast prototyping and testing of their highly evolving scientific ideas.

This is one of the reasons that Python language has become popular among researchers since the user operates with high-level abstractions, while hiding most of the low-level commands [2]. Hence, the scientist can concentrate on algorithm development rather than having to think about technical programming details. Python can be used in synchrotron X-ray imaging beamlines in several parts of the tomography pipeline from set up alignment to image acquisition and from data elaboration to reduction.

However, Python still cannot fit into many applications since, unlike C language, it has insufficient computational performance in many use cases. Recently, a trend has been adopted to mix the benefits of Python and C languages through hybrid frameworks. The design of these solutions keeps Python in the user front end, while modules that optimise the performance are implemented in C and work in the background. For this purpose, binding libraries have been introduced that wrap low-level codes and use them with Python code such as SWIG [3] and Cython [4].

Several commercial software offering analysis tools for volume images have been available since many years, such as, e.g., VGStudio [5], Avizo and Amira [6], MAVI [7], just to mention a few. Publicly available libraries have been developed by the community (e.g., DIPlib [8], ITK [9], PoreSpy [10]) and some of these also offer integration with Python. Research code tailored for specific analyses also exist (e.g., the 3DMApackages [11], Blob3D [12], Quant3D [13], iMorph [14]). In this context, Pore3D was conceived more than a decade ago by leveraging the experience of synchrotron users performing high-resolution X-ray micro-CT imaging. With this in mind, Pore3D was released and a first publication about it was presented in 2010. At that time, a Python wrapper was not considered basically due to the limited maturity of the Python platform and the related community. Nowadays, we believe Python support has become essential.

In this paper, we introduce PyPore3D, an open source software framework developed by the SYRMEP collaboration [15] at Elettra Sincrotrone Trieste, the Italian synchrotron radiation facility (Basovizza, Trieste). PyPore3D addresses the processing and analysis of 3D images of porous and/or multiphase media. These types of materials have been gaining increasing interest, especially with the development of multiscale and multimodal 3D and 4D imaging techniques ([16]). Accordingly, the development of efficient tools that support this type of study are highly demanded to explore microstructural properties in order to optimise manufacturing procedures or for engineering performance tuning [17,18], as well as to interpret and model phenomena in Earth science (see, e.g., LaRue et al., [19]; Mancini et al., 2020 [19]) or investigate biomedical specimens ([20,21,22,23]).

The goal of the proposed software code is to provide an efficient solution to 3D morpho-textural analyses that can be easily performed on commodity hardware. This solution is designed such that it combines the simplicity of Python language and the performance of the C one, while being easy to customise. Besides, it can also be integrated with the analytical approaches that are highly adopted by the current scientific community, including data science, machine learning and visual analysis tools. It is worth mentioning that the current version of PyPore3D does not include built-in 2D/3D visualisation modules. However, PyPore3D, in principle, can be integrated with a third party visualisation and analysis software that supports python (e.g., Avizo [6], Dragonfly [24] and imageJ [25]).

In this paper, we demonstrate how the software is used to meet various requests addressed by users of the SYRMEP beamline at Elettra as well as inspiring a possible customisation of similar scientific questions at other synchrotron and lab-based CT facilities.

## 2. Software Architecture

The PyPore3D package was developed for Linux and tested on Windows. In the following application examples, the reported results were generated on an Ubuntu 20.04.1 operating system (OS) installed on a workstation with an 8-Core processor and 16 GB of RAM. The performance results are shown for scripts that run on the Linux OS. The design of PyPore3D is based on two concepts:

1. Combining the benefits of open source tools and the simplicity of Python language with the powerful performance of C (especially with respect to integration with parallel computing and memory management);
2. Providing easy means for pipeline customisation through a modular architecture.

PyPore3D source code can be found in: https://gitlab.elettra.eu/aboulhassan.amal/PyPore3D (accessed on 13 June 2022).

**Figure 1:**
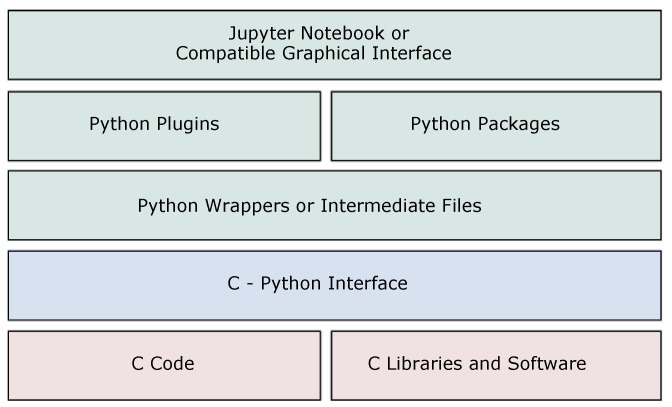
The Architecture of the PyPore3D package. The red blocks represent the Pore3D C code and illustrate that it can be extended with more C libraries. The green blocks represent the Python wrappers and show that it can be integrated with other Python packages and plugins. It can also be integrated with Jupyter Notebooks. The blue layer illustrates the binaries, which act as an interface that links the C and the Python layers.

The architecture of PyPore3D is illustrated in Figure 1.

The current package is customised by the specific know-how of the SYRMEP users collaboration while being highly modular. Therefore, it can inspire further customisation as well. As shown in Figure 1, the proposed architecture is composed of several conceptual layers, which interact together while enabling future extensions in both C and Python. In the current architectures, we implemented a layer between C libraries and Python code to enable the development of efficient computation modules in C code, while keeping the interface and inheriting the simplicity and scalability of Python. The details about the layer implementation are discussed in the following sections.

### 2.1. Pore3D C Core and C Libraries

The lowest layer represents the C code that is integrated with the PyPore3D pacakge. This layer currently includes the Pore3D C Core library proposed by Brun et al. [26,27], as the current package is building upon it. Pore3D provides efficient implementation of several commonly used filters and statistical analysis modules while supporting OpenMP extensions in many parts. Therefore, whenever C modules are required, they can also be extended in the C layer demonstrated in Figure 1, while benefiting from the C-Python Interface in the current architecture to easily integrate with Python code. In this layer, new C code can also be implemented in pure C code. In general, any C code will be integrated with the C-Python interface discussed next.

### 2.2. C-Python Interface

The C and Python packages are bound together through the C-Python interface. This interface is composed of cross platform scripts that transform the C code into binaries. The binaries are then called in intermediate Python files using the SWIG tool [28]. SWIG is a free software development tool that connects programs written in C and C++ with a variety of high-level programming languages. SWIG is used with different types of target languages, including common scripting languages such as Python. This library is typically used to parse C/C++ interfaces and generate the ’glue code’ required for the above target languages to call the C/C++ code.

### 2.3. Python Wrappers

The Python wrappers involve pure Python code developed specifically to wrap Pore3D in Python. These wrappers call the C-Python functions generated by SWIG. Besides, new code is developed to encapsulate them into more concise Python libraries that are easy to use and extend. For example, the new code includes help functions and function argument validations.

### 2.4. Python Plugins and Packages

Once the Python wrappers are called, they can be integrated with other Python toolkits. In the current library, we use intermediate files to communicate with different state-of-the-art packages such as Simple ITK [18] for multi-dimensional image analysis and Plotly [29] for advanced visualisation. Intermediate files are used for this purpose, which simply means that we internally save data and read them with the specialised readers of each package. Similar to the lower C layer, any new Python code can be implemented in this layer, which can also be totally independent from the Pore3D library.

### 2.5. Jupyter Notebook or Compatible Graphical Interface

The C code binaries are encapsulated into newly developed wrappers, which can be called easily from any Python editor or GUI, such as Jupyter Notebooks used in the current case studies.

## 3. Application Examples

In this section, the main functionalities of the PyPore3D Framework are applied on examples of image analysis of different materials. There is no unique protocol for the extraction of quantitative information from 3D images since it highly depends on the data type and specific application.

**Figure 2:**
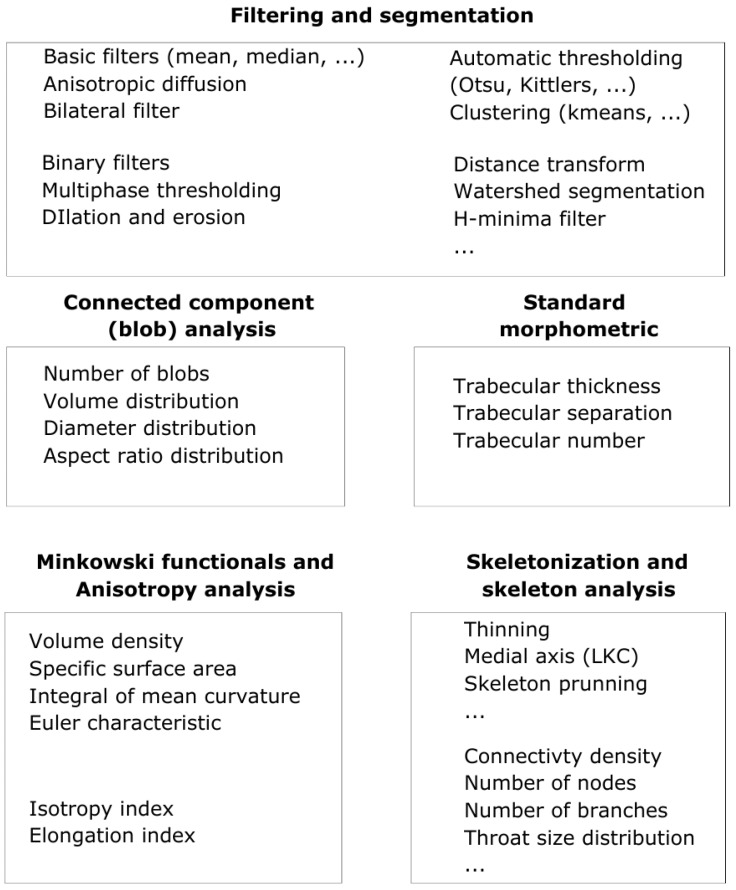
PyPore3D module architecture for quantitative analysis of 3D images. The modules used in the example applications are grouped in the figure.

The PyPore3D design takes into account the need for flexibility in data processing and analysis by providing simple and flexible scripting tools for the required protocols. The user can follow his own strategies having a full control of the parameters of the applied algorithm guided by the documentation. The main functions used in the current examples are illustrated in Figure 2. In the following case studies, the main functionalities of the Pore3D library are illustrated through several examples of analysis of X-ray images. The data have been collected at Elettra, using synchrotron radiation at the SYRMEP beamline [15,30].

Jupyter Notebooks that implement the examples in this section can be downloaded from: https://gitlab.elettra.eu/aboulhassan.amal/PyPore3D (accessed on 13 June 2022).

### 3.1. Statistical and Topological Pore Analysis

The parametrisation of the morphology of objects in the 3D domain that constitute natural and artificial materials (minerals, amorphous phases, voids) is a key factor for their description, study and interpretation. Applications encompass biomedical and materials science studies and all the fields of geology including petrology, volcanology, sedimentology and related fields. In fact, retrieving the real morphology of objects reduces the uncertainty inherent to the extension of the 2D measurements to the third dimension by means of stereological methods (e.g., Stroeven et al., 2009 [31]; Baker et al. 2012 [32]). A large number of papers has proved the benefits of a 3D characterisation of the phases composing rocks. For example, morphological characterisation of bubbles and minerals in volcanic products provide strong tools to reconstruct the regime of magma rising through dykes (Lanzafame et al., 2017 [33]), and the investigation mechanisms and timing of magma degassing (Lield et al., 2019 [34]). Moreover, recent advances in situ and real-time studies (4D CT) have demonstrated the necessity of accurate methods for monitoring the evolution, in terms of geometrical properties, of the phases composing rocks. An example is discussed by Voltolini et al. [35]. The evaluation of changes in number, volume and shape of crystals and vesicles deepens our comprehension of their nucleation and growth from silicate melts (Kudrna Prašek et al., 2018 [36]; Arzilli et al., 2019 [37]; Dobson et al., 2020 [38]; LeGall et al., 2021 [39]).

In the current section, we demonstrate how PyPore3D is used for pore statistical and topological analysis. Testing of the software was performed on image stacks obtained by synchrotron radiation computed microtomography (SRμCT) in propagation-based phase-contrast mode [40] of four rock samples with volcanic and sedimentary nature.

Sample SSK36 is a basaltic andesite lava rock from the effusive activity of Skaros volcano (Santorini, Greece—Lanzafame et al., 2020 [41]). Samples FL1 and TR2 were collected from two lava flows (basaltic trachy-andesite and trachy-andesite, respectively) belonging to the Ellittico volcanic sequence of Mount Etna (Sicily, Italy—Lanzafame et al., 2021 [41]). SC1 is a quartzarenitic xenolith found among the recent volcanic products of Mount Etna (Lanzafame et al., 2018 [42]). These samples were selected on the basis of their textures, characterised by a variable vesicularity with bubbles of different amount, size and degree of connectivity. Their presence in these rocks is related to the exsolution of magmatic water (FL1, TR2 and SSK36) or to gas migration processes occurring at sub-volcanic conditions (SC1).

All the SRμCT experiments were performed at the SYRMEP beamline of Elettra. The tomographic reconstruction was performed by using the Syrmep Tomo Project (STP) software suite (Brun et al., 2017 [26]). The experimental conditions adopted for each sample and tomographic reconstruction details are described in Lanzafame et al. [41,42,43]. A summary of the experimental parameters is reported in Table 1. More details about data treatment and interpretation are provided in the cited references.

**Figure 3:**
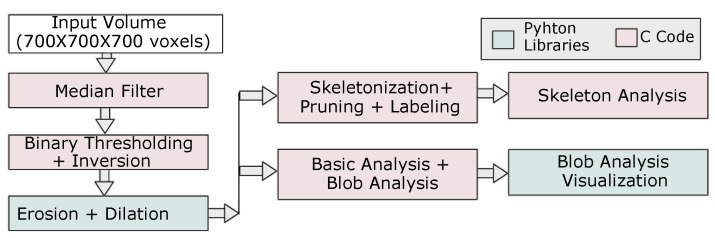
Processing and analysis protocol adopted for the volcanic data sets highlighting modules implemented in Python and modules implemented in C.

The adopted processing and analysis protocol is illustrated in Figure 3.

As shown in Figure 3, a combination of both C and Python packages is applied. This was implemented in a Jupyter Notebook and is reported in the gitlab link: https://gitlab.elettra.eu/aboulhassan.amal/PyPore3D (accessed on 13 June 2022).

First, a 3D Median filter was applied to the selected volume of interest (700 × 700 × 700 voxels). Then, the pores within this volume were segmented using the 3D automatic Otsu method [44] followed by the application of erosion and dilation morphological filtering. In this protocol, we used the binary dilation and erosion filters [45], as implemented in the Simple ITK library, while the Otsu method has been implemented in C. The method parameters are illustrated in Table 2.

**Figure 4:**
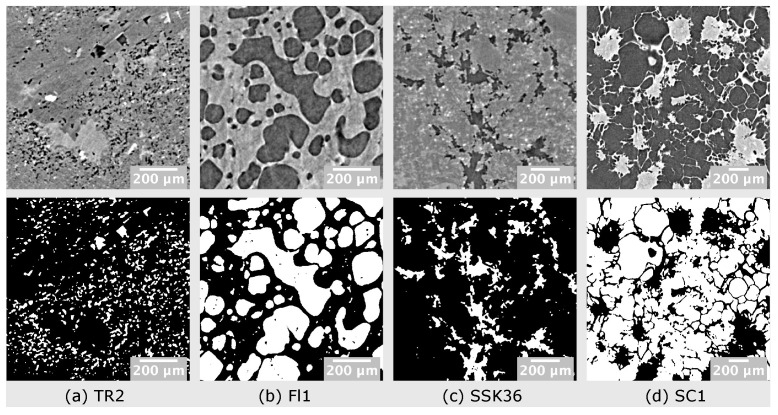
Raw and resulting processed images visualised using the freeware imageJ [25]. The upper row shows portions of reconstructed axial slices while the lower one shows the corresponding extracted pore phase after applying 3D median filtering, 3D automatic Otsu thresholding, erosion and dilation morphological filters.

In Figure 4, reconstructed virtual sections and the resulting processed images are shown for every data set sample.

#### 3.1.1. Basic Analysis

From the segmented volume, users can perform the Basic Analysis, which denotes the computation of the the basic identifiers that can be extracted. Examples of basic identifiers include the Isotropy (I) and Elongation (EA) indices. The I index is computed according to the mean intercept length method [46], while EA is the measure of the preferred fabric orientation [47]. In addition, basic Minkowski functionals are computed [48], which involve:

- Volume density (VV): quantifies the sample porosity. This is computed by the percentage of object voxels with respect to the considered volume.
- Specific surface area (SV): is a measure of the surface of the object with respect to the total volume.
- Mean curvature (MV): is an index of the dominance of convex or concave shapes.
- Euler characteristics ($\kappa_{v}$): is an index for the connectivity of the object network [49].

The results of the Basic Analysis are illustrated in Table 3. The average computational time for the Basic Analysis is 17 seconds on the given hardware specifications.

#### 3.1.2. Connected Components (Blob) Analysis

**Figure 5:**
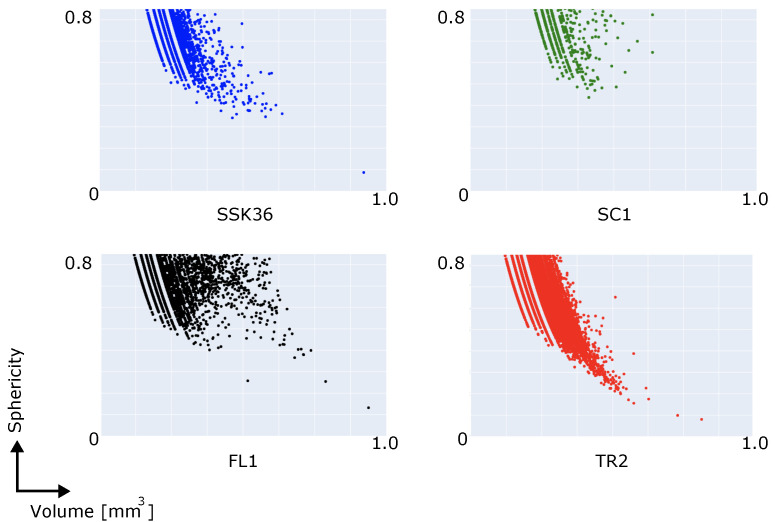
Results of the Blob Analysis of the vesicles-discussed in Table 2—showing the trends depicted by binary relations between the volume of each identified blob and sphericity (defined as the ratio between the diameter of the maximum inscribed sphere of each identified blob and the diameter of a sphere with the same volume as the blob). The figure is represented as a scatter plot. All the trends show a decrease in the degree of sphericity with increasing volume, indicating the tendency of vesicles to become more deformed as their size increases. On average, trends indicate a marked higher size of the vesicles in FL1 and SC1 samples being also characterised by a higher sphericity with respect to SSK36 and TR2 samples. This reflects the nature of the samples: FL1 and SC1 show a foamy texture, due to a high presence of glass and gas phases during their formation; SSK36 and TR2 are instead lava rocks featuring a highly crystalline or microcrystalline texture in which the gas phase was present in lower amounts compared to the foamy rocks.

In this section, we illustrate an example application of the (*Blob Analysis*) module. This analysis is conducted on the same data sets presented in Section 3.1 and provides indicators about connected component (blob) features such as the shape and volume of the blobs [27]. The result of theBlob Analysis module involves thousands of multi-dimensional points. Analysing all these points is a complex task, and hence, the selection of a method to properly visualise the results is part of the data analysis pipeline. In this example, we visualised the obtained results using Plotly [29], which is one of the state-of-the-art Python libraries. In the proposed case study, Plotly is integrated directly with the protocol script; similarly, other Python libraries can be integrated as well. An example of the Blob Analysis visualisation is shown in Figure 5.

#### 3.1.3. Skeleton Analysis

**Figure 6:**
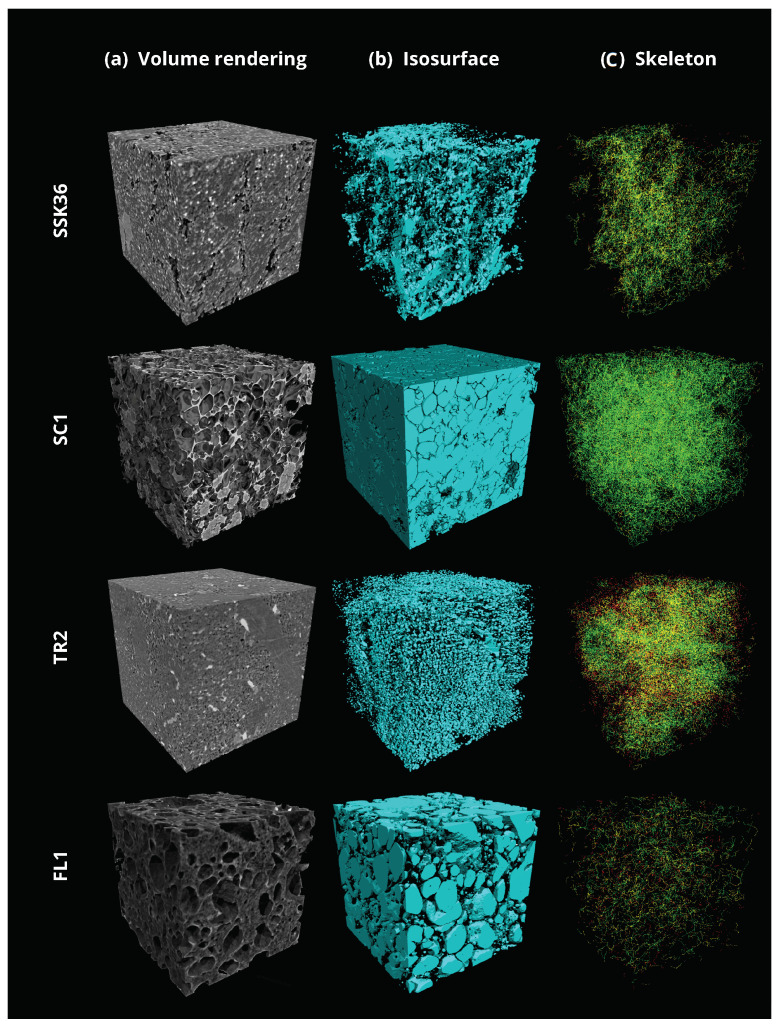
Results of microtomographic reconstruction and image processing on the same volume of interest (VOI, size = 700 × 700 × 700 voxels) for the four investigated samples. (**a**) 3D renderings of raw data as obtained after tomographic reconstructed. (**b**) Corresponding isosurface renderings of the segmented vesicle phase. (**c**) Results of the skeletonisation analysis of the vesicle network representing node-to-node (green), node-to-end (yellow) and end-to-end (red) branches.

The next step of the applied protocol is represented by the Skeleton Analysis. From the binary (filtered and segmented) data sets, the skeleton structure is computed. As shown in Figure 3, the skeletonisation is implemented inside the Pore3D package. This package offers several skeletonisation algorithms [50,51,52], as well as pruning methods [53], for this purpose. The algorithm can be selected by the user depending on the specific microstructural features of the investigated sample and on the information to be extracted. Results of the vesicle phase segmentation and related Skeleton Analysis are shown in Figure 6.

In this analysis, we applied the LKC skeletonisation algorithm, which was proposed in [50]. The results of the skeletonisation procedure are illustrated in Table 3. The average computational time for the Skeleton Analysis is 3700 seconds on the given hardware specifications.

### 3.2. Analysis of Multiphase Materials

In this section, we show an example of the protocol applied to characterise a multi- phase material. Multiphase materials are commonly studied by the scientific community. They target phases corresponding to components with different chemical compositions, including mass density, structural or morphological properties. These components can correspond to different gray levels in the X-ray tomograms. In many applications, it is required to separate these phases in order to quantitatively analyse each of them.

The sample used in the current case study is an Ottawa sand in brine at 0 degrees C. SRμCT data were collected at the 8.3.2. beamline Advanced Light Source using 25 keV monochromatic X-rays, with an exposure time of 550 ms for each of the 1441 projections, using a Peltier-cooled cell during a in situ experiment aimed at studying permafrost evolution. Pixel size was 3.44 microns and the sample-to-detector distance was 5 cm. A 1.5 M KI brine was used both as a contrast agent and to reach a realistic salinity level and provide absorption contrast in the data set.

**Figure 7:**
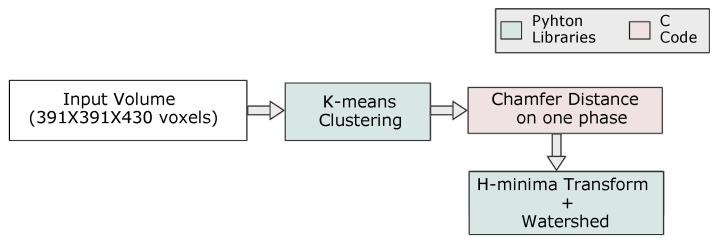
The protocol used for segmentation and watershed filtering.

In the current example, we show how the use of the Simple ITK functions—integrated with PyPore3D—can generate correct segmentation. The Simple ITK library implements several state-of-the-art segmentation methods such as k-means and multiple thresholding methods. We integrated these functions with the current PyPore3D wrappers of the Pore3D C code to allow users to integrate the C code in a simple script whose applied protocol is illustrated in Figure 7.

Multiphase segmentation is explored in the current application using the k-means clustering algorithm [44]. The implementation is integrated with PyPore3D, where the user only needs to determine the number of classes.

**Figure 8:**
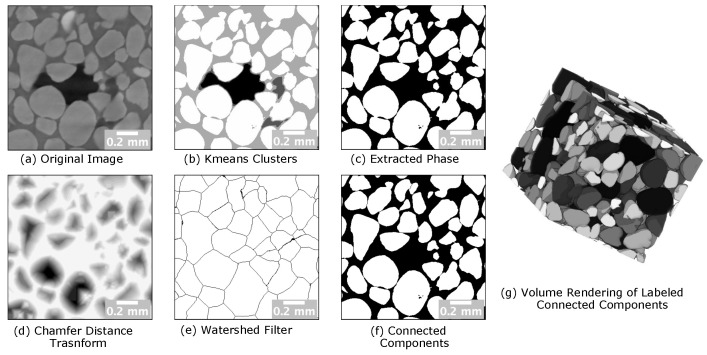
Example of applying connectivity component analysis. (**a**) The original image of the "sand in brine" data set. (**b**) Applying k-means clustering with 4 classes to explore phases of interest. (**c**) Binary image representing the extracted phase of interest using a masking. (**d**) Chamfer distance transform with H-Minima filter of threshold = 4 (**c**). (**e**) Morphological Watershed Filter applied (**d**). (**f**) Extracting connected components from the Watershed filtered image. (**g**) Volume rendering representing the labelled connected components (Blobs); 286 objects were found. The light gray phase corresponds to the sand grains (quartz), the dark gray phase corresponds to the brine, and the darkest gray corresponds to air.

The detailed adopted protocol is illustrated in Figure 7, while in Figure 8, we report the results of segmentation performed using four classes. From Figure 8, we can verify that the sample contained three phases of interest only. In the same figure, we also show the results of Watershed filtering [54]. Watershed filtering is one of the classical algorithms used to addresses several challenges. For example, for data involving blob shapes, the segmentation should be able to produce a binary image having each blob represented by a set of contiguous voxels, as the case of 3D data sets, including pores that are physically interconnected, while they should be considered semantically separated. Moreover, even if pores are not physically connected, they might appear connected in the 3D image due to a limited spatial and/or contrast resolution or due to the presence of artifacts in the images. To this purpose, scientists frequently use this method in combination with distance transform and masking [26]. Usually, it is needed to refine the results through either pre-processing of the original binary image by using morphological operators (dilation and erosion) or post-processing with a H-minima filter [55].

In Figure 8, we show how the Watershed Filtering is used to effectively separate the sand grains. The current Watershed algorithm used for this type of segmentation is based on the technique proposed in [56]. The results are generated by combining PyPore3D C modules, Python wrappers of Pore3D and Simple ITK Python modules. The performances of the algorithm in terms of computational time are reported in Table 4.

### 3.3. Morphometric Analysis

In this section, we investigate the implementation of the PyPore3D Morphometric Analysis module through an example of applications in biomedical data. This module can extract a series of analytical parameters, directly in the 3D domain, suitable for trabecular-like porous media such as the bone data depicted in the current case study.

The bone data collection was performed with the agreement from the local ethics committee and according to the 1975 Helsinki Declaration (revised in 2000). The donor was a female (96 years old, 1.63 height, dual-energy X-ray absorptiometry 0.939 g/cm^2^). The sample was cut using a bandsaw along the axial direction right below the lesser trochanter (approximately 10 to 12 cm section proximal to the femur head) and the specimen were then stored at −25 °C until scanning. The central femoral neck core acquired using the SRμCT at 5 μm^3^, in which the trabecular phase was present only, and thanks to the small isotropic voxel size (0.9 μm), the image field of view was focused on a single trabecula with the osteocyte lacunae becoming assessable.

Propagation-based phase-contrast SRμCT was used to obtain the 3D virtual reconstruction of the bone microstructure. The central core across the whole length of the femoral neck was imaged at the SYRMEP beamline of Elettra using a filtered (1.5 mm Si plus 1 mm Al) polychromatic X-ray beam corresponding to a mean energy of ca 27 keV. The detector used was a water-cooled, 16-bit, sCMOS macroscope camera (Hamamatsu C11440–22C) with a 2048 × 2048 pixels chip coupled with a GGG:Eu scintillator screen, 45 μm thick, through a high numerical aperture optics. The effective pixel size of the detector was set at 5.0 × 5.0 μm^2^, yielding a maximum field of view of about 10.2 × 10.2 mm^2^. The sample-to-detector (propagation) distance was set at 150 mm. For each scan, a set of 1200 projections was recorded with continuous sample rotation over a 180-degree scan angle and an exposure time per projection of 1.0 s. Each set of acquired raw images was processed using the STP software suite. A single-distance phase-retrieval algorithm was applied to projection images before CT reconstruction [57], setting the ratio between the real and imaginary parts of the complex refraction index of the material under investigation to 50.

**Figure 9:**
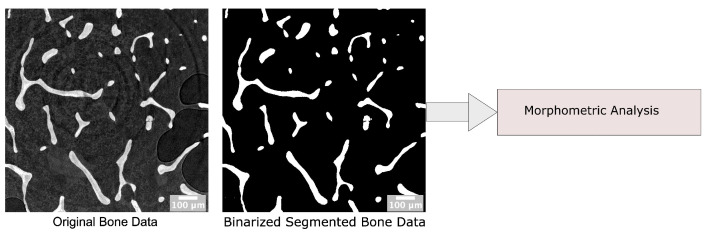
The protocol used for the application of the Morphometric Analysis on bone data. The analysis is done on the binary segmented data to separate the bone phase. The size of the volumetric data set is 1178 × 1178 × 200 voxels with an isotropic voxel size of 5.0 μm.

An example of data processing results are displayed in Figure 9.

The parameters computed in the Morphometric Analysis [58,59] module are defined as follows:

BvTv: The ratio between bone volume and total volume;
BsBv: The ratio between bone surface and bone volume;
TbN: The trabecular thickness;
TbTh: The trabecular separation of the solid phase objects;
TbSp: Trabecular number measuring the number of traversals across a solid structure.

The results of the analysis are reported in Table 5.

## 4. Conclusions and Future Work

In the previous sections, we have reported several examples of tomographic image processing and analysis modules that are commonly used in materials science characterisation through X-ray imaging techniques. We also presented different domains of application such as geological and biomedical samples. The examples explored several processing functions such as filtering, segmentation and skeletonisation. In parallel, we also reported the computational time needed for the different operations that imply various levels of complexity and, based on the hardware used, limitations in data size.

The corresponding performance in terms of computational time and data sizes, denotes the feasibility of running PyPore3D on modern commodity hardware with no need for special equipment. Some computations are more time-consuming such as skeletonisation for some data sets. However, there is room for further optimisation using parallel computing, for example, which benefits from mixing C and Python languages. Another aspect of performance is usability. The users can develop relatively complex analysis scripts with minimal programming effort. As we have discussed in the previous examples, the software is currently being tested on tomographic data sets of different specimens. For each data set, the user created a Python Jupyter notebook for every analysis protocol involving different modules from PyPore3D, SimpleITK Python wrappers, and plugins from other state-of-the-art Python packages such as Plotly.

PyPore3D gives the users full control on the analytical protocol to be adopted allowing mixing of various types of functions in one script with no special licenses or setups as well as making use of many Python packages to reduce the analysis workflow by including several functions in one script. This solution demonstrates a substantial improvement in increasing the beamtime efficiency in terms of obtained results, and its highly modular concept could inspire further customisation and extensions from developers. It also provides an easy way to exchange analysis solutions and foster collaborations within the user community by sharing Jupyter Notebooks.

In the future we plan to integrate advanced methods in the fields of machine learning and computer vision. These approaches can be employed to automate and fasten the segmentation and feature detection in large and complex data sets such as, for instance, in the case of dynamic tomographic acquisition (the so-called 4D CT). These domains are getting high importance in the scientific fields due to their power in automating the analysis and processing of big data. The related common tools currently developed by the scientific community are based on Python and PyPore3D enables the involvement of these tools in the relevant data reduction pipelines.

## Figures and Tables

**Table 1:** Experimental parameters used for the tomographic scan. The filter used for all samples was 1.5 mm of Si plus 1 mm of Al.

	TR2	FL1	SSK36	SC1
Sample to detector dist. (mm)	150	150	200	100
Pixel Size (μm)	1.37	1.37	1.8	2.6
N° of Projections	1800	1800	1800	1800
Exposure time per projection (s)	2	2	2	1

**Table 2:** Parameters of the PyPore3D protocol used on the samples: TR2, Fl1, SSK36 and SC1.

	TR2	FL1	SSK36	SC1
Median width	3	3	3	3
Automatic 3D single Otsu threshold	104	125	105	122
Erosion/Dilation width	3	3	3	3
LKC Pruning	5	5	5	5
Isotropic voxel size (μm)	1.37	1.37	1.8	2.6

**Table 3:** Results of the Basic Analysis and the Connectivity Density computed from the Skeleton Analysis modules performed on the data sets of TR2, FL1, SSK36 and SC1 samples.

	TR2	FL1	SSK36	SC1
$\kappa_{v}$ (mm^3^)	37,436	9842	1873	−2557
MV (mm^2^)	5585	611	593	60
SV (mm^1^)	43	29	18	27
VV	0.1	0.5	0.1	0.7
EA	0.08	0.08	0.11	0.03
I	0.89	0.89	0.85	0.85
Connectivity Density	5214	644	3273	3193

**Table 4:** Performance results of multiphase analysis.

Method	Time (s)
K-means (4 classes)	78
Inverted Distance Field	2
H-minima Filter	2
Watershed Filtering	16

**Table 5:** The table shows the results of PyPored3D of the Morphometric Analysis on bone data. The total computation time was 0.3 s.

Morphometric Indicator	Value
BvTv	0.1
BsBv (mm^−1^)	32
TbN (mm)	1.61
TbTh (mm)	0.06
TbSp (mm^−1^)	0.56

## Data Availability

All the data used in this section can be found in the Open/FAIR Elettra scientific data repository [60]. According to the restrictions imposed by Aix Marseille University and by the local ethics committee regarding patient data sharing, data could be made available upon reasonable request addressed to Monique Bernard (monique.bernard@univ-amu.fr) pending the signature of an MTA approved by Aix Marseille University.
